# Pharmacological Modulators of Small GTPases of Rho Family in Neurodegenerative Diseases

**DOI:** 10.3389/fncel.2021.661612

**Published:** 2021-05-12

**Authors:** William Guiler, Addison Koehler, Christi Boykin, Qun Lu

**Affiliations:** Department of Anatomy and Cell Biology, The Harriet and John Wooten Laboratory for Alzheimer’s and Neurogenerative Diseases Research, Brody School of Medicine, East Carolina University, Greenville, NC, United States

**Keywords:** Rho GTPases, pharmacological modulators, neurodegeneration, Alzheimer’s disease, cytoskeleton

## Abstract

Classical Rho GTPases, including RhoA, Rac1, and Cdc42, are members of the Ras small GTPase superfamily and play essential roles in a variety of cellular functions. Rho GTPase signaling can be turned on and off by specific GEFs and GAPs, respectively. These features empower Rho GTPases and their upstream and downstream modulators as targets for scientific research and therapeutic intervention. Specifically, significant therapeutic potential exists for targeting Rho GTPases in neurodegenerative diseases due to their widespread cellular activity and alterations in neural tissues. This study will explore the roles of Rho GTPases in neurodegenerative diseases with focus on the applications of pharmacological modulators in recent discoveries. There have been exciting developments of small molecules, nonsteroidal anti-inflammatory drugs (NSAIDs), and natural products and toxins for each classical Rho GTPase category. A brief overview of each category followed by examples in their applications will be provided. The literature on their roles in various diseases [e.g., Alzheimer’s disease (AD), Parkinson’s disease (PD), Amyotrophic lateral sclerosis (ALS), Frontotemporal dementia (FTD), and Multiple sclerosis (MS)] highlights the unique and broad implications targeting Rho GTPases for potential therapeutic intervention. Clearly, there is increasing knowledge of therapeutic promise from the discovery of pharmacological modulators of Rho GTPases for managing and treating these conditions. The progress is also accompanied by the recognition of complex Rho GTPase modulation where targeting its signaling can improve some aspects of pathogenesis while exacerbating others in the same disease model. Future directions should emphasize the importance of elucidating how different Rho GTPases work in concert and how they produce such widespread yet different cellular responses during neurodegenerative disease progression.

## Introduction

Rho GTPases are a subfamily of the Ras superfamily proteins and include the key classical proteins RhoA, Rac1, and Cdc42 of the over 20 that have been identified. In addition to critical functions in the nervous system, Rho GTPases regulate cellular behavior such as morphogenesis, survival, proliferation, membrane trafficking, adhesion, and transcriptional activation (Zegers and Friedl, 2014; Arrazola Sastre et al., 2020; Clayton and Ridley, 2020). Rho GTPases are found in all eukaryotic species and are highly conserved which underscores their importance for survival (Hall, 2012; Beljan et al., 2020).

**Figure 1:**
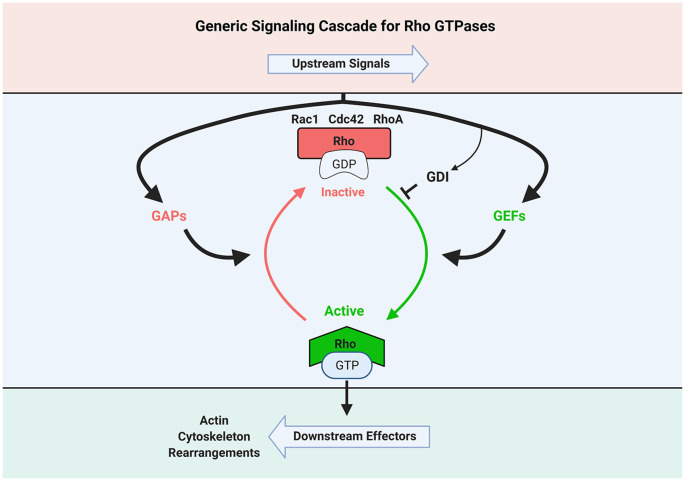
Schematic illustration of Rho GTPase activation and inactivation signaling cascade. GEF, Guanine nucleotide exchange factor; GAP, GTPase-activating protein; GDI, GTPase dissociation inhibitor. Created with BioRender.com.

These small G-proteins act as switches that trigger signaling to many molecular pathways by oscillating between inactive GDP-bound and active GTP-bound states (Figure 1). Thus, they transduce upstream signals to downstream effectors leading to numerous cellular processes including changes in actin cytoskeleton (Steffen et al., 2017).

The transition between active and inactive conformational states is highly regulated by guanine nucleotide exchange factors (GEFs) and GTPase-activating proteins (GAPs). Rho proteins are activated *via* GEFs through the exchange of G-protein-bound GDP to GTP, while they become inactivated by GAPs through GTP hydrolysis (Narumiya, 1996). It is important to note that while Rho GTPases often work in concert, their signaling cascades are unique and regulated by selective GEFs and GAPs.

Within the central nervous system (CNS), Rho GTPases have been implicated in nearly all steps of brain development (Zamboni et al., 2018). There was an early lack of studies examining the precise roles of these proteins in mammalian CNS *in vivo* (Heasman and Ridley, 2008) because mice with Rho GTPase inactivation often do not survive (Luo et al., 1996; Chen et al., 2000). More recent studies utilizing tissue or region-specific deletion have allowed for the roles of these diverse proteins to be uncovered within neurogenesis and neuronal maturation (Vaghi et al., 2014). Consequently, Rho family small GTPases have been revealed to play a key role in brain functions including learning and memory (Musilli et al., 2013).

Rho GTPases are involved in regulating the neuronal actin cytoskeleton, synaptic plasticity and dynamics of dendritic spines, neurotransmitter receptor clustering and induction of long-term potentiation (LTP; Jones et al., 2004; Auer et al., 2011; Vaghi et al., 2014; Hedrick and Yasuda, 2017; Zamboni et al., 2018). Due to Rho GTPase’s implication on learning and memory, it is not surprising that genetic alterations of these small G-proteins lead to disastrous consequences in the form of neurodegeneration. The implication of Rho GTPases in such disorders provides further evidence of their necessity in physiological functioning. Perhaps the most causal relationship with malfunctioning GTPase proteins and neurodegeneration can be implicated in familial Frontotemporal Dementia (FTD) and Amyotrophic lateral sclerosis (ALS), where the gene product of C9ORF72 presents a Rho GTPase GEF activity. C9ORF72 may function as a Rho GTPase modulator that controls actin cytoskeleton and autophagy with endocytosis (Iyer et al., 2018). In addition, mutations of ALS2 have been linked to ALS encoding the protein alsin2, which carries a diffuse B cell lymphoma (Dbl) homology/pleckstrin homology domain (termed DH/PH) and presents RhoGEF properties (Hadano et al., 2001).

Increasing evidence supports a role for Rho GTPases in various neurodegenerative diseases (DeGeer and Lamarche-Vane, 2013; Stankiewicz and Linseman, 2014). For example, in Alzheimer’s disease (AD) brain, the overall RhoA levels are found to be reduced, while remaining RhoA co-localized with hyperphosphorylated tau in neurofibrillary tangles (Huesa et al., 2010). Additionally, RhoA expression is decreased within synapses but increased in degenerating neurites of amyloid precursor protein (APP) over-expressing transgenic mouse brains (Huesa et al., 2010). Most striking is that the activity of RhoA/ROCK enhances Aβ production by the secretase-dependent APP cleavage (Zhou et al., 2003). Interestingly, human neuroblastoma cells exposed to Aβ show enhanced activation of RhoA and diminished activation of Rac1 (Petratos et al., 2008). In AD, patient brains show diminished expression of Rac1 (Zhao et al., 2006). Similarly, Kalirin-7, a Rac1 GEF, is down-regulated in AD hippocampal tissues as examined *via* Kalirin-7 mRNA and protein levels (Youn et al., 2007). The p21 activated kinase (PAK), a Rac1 effector, shows a decrease in localization in hippocampal sections but an up-regulation in intraneural levels in patients (Zhao et al., 2006). PAK also shows increased activity following Aβ42 exposure in hippocampal neurons (Mendoza-Naranjo et al., 2012). Although Cdc42’s role is less understood in neurodegeneration, the Cdc42 GAP, NOMA-GAP, has been implicated in AD. A stark reduction in cortical thickness is a hallmark of AD, and NOMA-GAP has been shown to modulate cortical thickness and neuronal dendritic branching during development (Rosario et al., 2012). If NOMA-GAP is suppressed, Cdc42 becomes hyperactivated and leads to an oversimplification of dendritic arborization. A reduction of Cdc42 levels can mediate these results (Rosario et al., 2012). On the other hand, the alteration of Cdc42 signaling is inferred in the studies that showed intersectin (ITSN), a Cdc42GEF, is highly induced in AD and Down syndrome (DS; Hunter et al., 2013).

Rho GTPases are also highly implicated in Parkinson’s disease (PD) pathology. One study characterized a direct interaction between a RhoGEF for Rac GTPases Kalirin-7 and synphilin-1 and found that Kalirin-7 expression increased the likelihood of synphilin-1 aggregated Lewy bodies (LB) to be degraded (Tsai et al., 2012). Mutations in leucine-rich repeat kinase 2 (LRRK2) have been regarded as the most common genetic cause of late-onset PD. Chan et al. (2011) identified that LRRK2 activates Rac1. Conversely, mutant LRRK2 does not cause activation. Such lack of Rac1 activation leads to neurite retraction (Chan et al., 2011). In PD, inhibition of ROCK delays the onset and extends survival in mice administered with MPTP (Tönges et al., 2012). Moreover, ROCK inhibition blocked microglia from removing dopaminergic neurons in the MPTP-treated mice (Barcia et al., 2012). As such examples showed, it is clear that Rho GTPases are implicated in a variety of neurodegenerative diseases.

### Aims

This article will provide an overview of the different categories of pharmacological modulators of Rho GTPases and recent literature on potential therapeutic options for Alzheimer’s disease (AD), Parkinson’s disease (PD), Amyotrophic lateral sclerosis (ALS), Huntington’s disease (HD), Multiple sclerosis (MS), among others. While it is impossible to cover all regulatory elements of Rho GTPase signaling cascades, this review will present some examples to demonstrate the importance of Rho GTPases in the drug discovery process for neurodegenerative diseases.

### Small GTPases of Ras Superfamily

Early studies showed that Ras superfamily GTPases control toxic peptides, such as Aβ42 in amyloid plaques and phosphorylated tau of neurofibrillary tangles, *via* the MAPK pathway (Gärtner et al., 1999). They also induce dendritic spine and synapse loss, which are major AD hallmarks, and Ras controls the L-DOPA-induced dyskinesia in PD as well (Arrazola Sastre et al., 2020).

Nearly universal components of signaling pathways, the Ras superfamily can be divided into five major subfamilies—Ras, Rho, Rab, Arf, and Ran (Qu et al., 2019). These small GTPases are activated in response to extracellular stimuli, and they help regulate cytoplasmic signaling networks. The largest subfamily, Rab, is related to membrane trafficking in signaling pathways, including the endocytic and secretory pathways. While there are fewer studies on Arf and Ran subfamilies in neurodegenerative diseases, the Rab subfamily is also implicated in AD but is reviewed elsewhere (Qu et al., 2019). The Rho subfamily, with crucial roles in cytoskeletal remodeling in neurodegeneration, is the focus of this review.

### Rho GTPase Subfamily

It is widely understood that the regulation of actin polymerization plays a key role in neuronal morphological changes seen in neurodegenerative disorders. The Rho subfamily of small GTPases are the most prominent regulators of actin reorganization. The commonly studied classical members of the Rho subfamily include Rac1 (Ras-related C3 Botulinum toxin substrate 1), RhoA (Ras homolog gene family member A), and Cdc42 (cell division cycle 42; Aguilar et al., 2017a). While the classic Rho GTPases play important roles in actin cytoskeletal regulation, Miro1 (RhoT1) and Miro2 (RhoT2) are atypical Rho-like GTPases involved in modulating mitochondrial homeostasis and apoptosis and are strongly implicated in neurodegenerative diseases such as PD (Fransson et al., 2003; Stephen et al., 2015).

Cdc42 is a small GTPase that participates in progenitor cell formation, as well as their differentiation into neurons, which gives it a crucial role in neurogenesis (Vadodaria et al., 2013). The activation of Cdc42 stimulates axonal growth, spine formation, and dendritic branching. The presynaptic activation of Cdc42 leads to an effect similar to the effects of electrical activity that promotes synaptic maturation and plasticity (Shen et al., 2006). The Cdc42 regulation of neurogenesis is activated by different but selective GEFs, including ITSN.

More specifically, ITSN is involved in dysregulation of endocytic trafficking, an early event observed in patients with AD or DS. ITSN is identified in unbiased gene profiling as one of the most highly induced genes in AD and DS patients (Hunter et al., 2013). Both ITSN and Cdc42 are essential for neuronal spine development and neuronal survival. ITSN-1 overexpression alters Cdc42-mediated endocytosis, potentially contributing to AD pathology (Arrazola Sastre et al., 2020).

Cdc42 has been shown to cause significant deregulation in AD leading to cytoskeletal alterations (Mendoza-Naranjo et al., 2007, 2012). Indeed, increased levels of Cdc42 have been reported in AD patients in select neuronal populations (Zhu et al., 2000; Aguilar et al., 2017b). However, other studies reported no major changes for Rac1, Cdc42, or PAK in AD and APP transgenic mice (Huesa et al., 2010).

Rac1 shares many aspects in signaling as Cdc42, and also plays an important role in axonal growth, spine formation, and dendritic branching as well as neurogenesis (Vadodaria et al., 2013). This member is involved in reduction of Aβ42 levels and controls tau phosphorylation in AD (Borin et al., 2018). Rac1 leads to reduction of alpha-synuclein (α-Syn) and rescues neurite retraction caused by G2019S LRRK2 in PD (Chan et al., 2011). Many effectors have been identified for Rac1, including PAK, the PI3K/PDK/nPKC axis, NOX, and the JNK pathway. Recent evidence suggests the mRNA expression level of Rac1 was down-regulated in the entorhinal cortex (EC) of AD brains. Furthermore, this down-regulation resulted in behavioral deficits and neurodegeneration (Kikuchi et al., 2020). Rac1 has also been identified in Autism-Spectrum Disorder (ASD) and Intellectual Disability (ID) animal models with Rac1 being implicated in many genetic abnormalities in ASD/ID (Tian et al., 2018).

RhoA is the member of the Rho subfamily that is correlated with the formation of actomyosin contractile fibers (Arrazola Sastre et al., 2020). Specifically, changes in RhoA localization have been linked to neurodegeneration seen in AD (Huesa et al., 2010). Studies completed with APPSwe Tg2576 mouse model expressed reduced levels of RhoA in synaptic ends and increased levels in dystrophic neurites (Huesa et al., 2010). Additionally, RhoA promotes focal adhesion.

It is well-known that RhoA often acts in opposition to Rac1 and Cdc42 to control protein processing and trafficking as well as synaptic remodeling (Tashiro et al., 2000; Salloum et al., 2020). Unlike Rac1 or Cdc42, RhoA activation is inhibitory, rather than stimulating neuronal processes and branching. Therefore, broad inhibition of RhoA signaling is widely believed to be a viable approach to curtail neurodegenerative diseases as we will discuss in more detail. On the other hand, a recent study indicated an interesting link between microglia that lack RhoA and neurodegeneration. The ablation of RhoA in microglia has produced AD-like pathology in mice, and the absence of RhoA led to synapse loss and memory deficits (Socodato et al., 2020).

As aforementioned, the RhoA/ROCK pathway has been implicated in several neurodegenerative diseases due to dysregulation. Drugs such as Nonsteroidal anti-inflammatory drugs (NSAIDs) and statins have been shown to act likely through the Rho/ROCK signaling pathway directly or indirectly and have been proposed widely as potential therapeutic targets (Bolognin et al., 2014). Huesa et al. (2010) investigated the expression of Rho GTPases in AD patients and mouse models of AD (i.e., Tg2576) and found significant RhoA mis-localization. These studies corroborated with the finding that RhoA expression was decreased at the synapses but showed an increase in dystrophic neurons (Petratos et al., 2008). Conversely, increased RhoA levels have been shown to impair LTP and learning while exhibiting neurodegeneration (Aguilar et al., 2017b). With neurodegeneration occurring at both low and high RhoA expression levels, homeostatic balance of RhoA signaling may be crucial to physiological functioning. Clearly, the regulation of RhoA/ROCK pathway is more complex in AD than previously thought and should be explored further to elucidate the precise modulatory mechanisms.

### Signaling Steps

It is well known that GEFs activate Rho GTPases and GAPs terminate their signal. Upwards of 80 GEFs and 70 GAPs have been discovered with most of them being expressed in the human brain. Although much of their function is not yet understood, recent studies have allowed for elucidation for how these regulatory proteins modulate synaptic plasticity (Ba and Nadif Kasri, 2017). RhoGAP and RhoGEF families have been shown to become activated by extracellular signals such as neurotransmitters (i.e., glutamate), neurotrophins, and adhesion molecules (Park et al., 2010; Martin-Vilchez et al., 2017).

It is important to note that in addition to RhoGAPs and RhoGEFs, other proteins can also influence Rho GTPase signaling. For example, neurotrophins stimulate neurite outgrowth by inhibiting RhoA activity, whereas myelin-derived proteins activate RhoA and thereby inhibit growth. This process is modulated by the direct sequestration of the Rho GDP dissociation inhibitor (RhoGDI) by p75 neurotrophin receptor (p75^NTR^) to activate RhoA (Yamashita and Tohyama, 2003; Harrington et al., 2008). Likewise, Kim et al. (2008a,2008b) demonstrated that δ-catenin, a neural member of the p120^ctn^ subfamily of armadillo proteins, decreased RhoA activity by sequestering p190RhoGEF. As such, δ-catenin and other scaffolding proteins may also act as GDIs that modulate GTPase and GEF interactions. Thus, it is necessary to envision Rho GTPase signaling as having multiple protein modules leading to potential activation or inactivation (Figure 1).

In fact, the activation of glutamate receptors leads to the phosphorylation of Kalirin-7 (Xie et al., 2007). Kalirin-7, for example, has been determined to be a critical regulator of dendritic spine growth (Penzes and Jones, 2008). Other GEFs, such as the Rac1-specifc GEF Tiam1 (T-lymphoma invasion and metastasis 1) also contribute to spine development (Tolias et al., 2011). For GAPs, α1-chimaerin is involved in regulating dendritic spine morphogenesis (Valdez et al., 2016). Bcr (Breakpoint cluster region) and Abr (active Bcr-related), both Rac1-GAPs, modulate excitatory synapses in the hippocampus (Oh et al., 2010). Lastly, RhoGAP oligophrenin-1 has been discovered as a key regulator of synapse development (Ba and Nadif Kasri, 2017).

**Figure 2:**
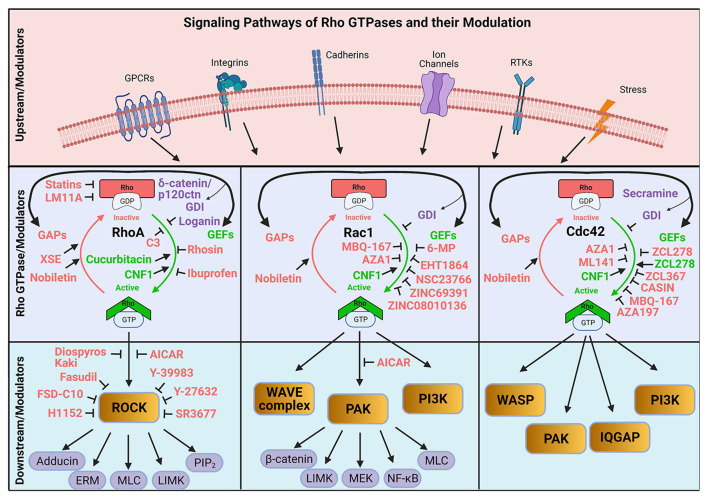
Schematic illustration of the modulation of Rho GTPase activation and inactivation by pharmacological approaches. GEF, Guanine nucleotide exchange factor; GAP, GTPase-activating protein; GDI, GTPase dissociation inhibitor; XSE, *Xanthoceras sorbifolia* extract; RTK, Receptor tyrosine kinases; PAK, Serine/threonine p21-activated kinases; PI3K, Phosphatidylinositol 3-kinase. Created with BioRender.com.

Thus, the activation of RhoGAPs and RhoGEFs then lead to neuronal morphogenesis, dendritic spine growth, and synaptic plasticity. These regulatory proteins serve as signaling mediators that, when activated by extracellular signals, induce Rho GTPase activity and subsequent actin cytoskeleton rearrangement enabling the neuronal functions needed for learning and memory (Martin-Vilchez et al., 2017). As such, the various signaling pathways associated with Rho GTPases—RhoGAPs, RhoGEFs, and Rho GDIs—represent viable drug targets for neurodegenerative diseases (Lu et al., 2009; Aguilar et al., 2017b). Here, we will briefly review the currently available pharmacological modulators of Rho GTPases (Table 1) and their proposed interactions with the Rho GTPase signaling pathways (Figure 2).

**Table 1:** Pharmacological modulators of Rho GTPases and their potential applications.

Treatment	Model in which it was tested	Effects	Reference examples
	SOD1-G93A mouse model for (ALS)	Delay motor symptoms thereby improving survival and protection of spinal cord motor neurons.	Takata et al. (2013)
	MPTP-lesioned mice (PD)	Improved motor behavior by preserving nigrostriatal fibers.	Tönges et al. (2012)
	Prnp.αSyn.A53T-expressing transgenic mice (PD)	Improved motor and cognitive functions	Tatenhorst et al. (2016)
	APP/PS1 mice (AD)	Increased dendrite branching	Couch et al. (2010)
Fasudil (ROCK1 and ROCK2 inhibitor)	EAE mouse model (MS)	Inhibits iNOS expression, decreases chemokines in astrocytes, reduces permeability of BBB, balance macrophages/microglia functions, and promotes remyelination.	Yan et al. (2019)
	BV-2 Murine microglial cell line treated with LPS to induce inflammation	Reduce NO production and phagocytic activity of reactive microglia and reduce neuroinflammatory response.	Scheiblich and Bicker (2017)
	Human Ntera2/D1 precursor cells differentiated into neurons (hNT2)	
*Xanthoceras sorbifolia* extracts	SD rats injected with Aβ_25-35_ aggregates	Improve cognition and ameliorate dendritic spine density deficit.	Li et al. (2016)
Y-39983 (selective ROCK inhibitor)	EAE mouse model (MS)	Suppress EAE clinical symptoms and prevent relapse after disease onset.	Gao et al. (2013)
CNF1, activator of RhoA, Rac1 and Cdc42	Lesion induced Wistar rats (PD)	Increase the size/number of cellular processes.	Musilli et al. (2016)
	SH-Sy5Y cell line treated with 6-OHDA (PD)	Preserve cell viability, counteracts oxidative stress, and triggers autophagy.	Travaglione et al. (2020)
Flavonoids from Diospyros kaki leaves (RhoA regulator)	APP/PS1 4-month-old mice (AD)	Improve learning and memory while decreasing RhoA activity.	Ma et al. (2018)
	OBX mice	Improves memory impairment	
	Aβ-infused rats	Protects against Aβ-induced memory deterioration.	
	SAMP8 mice	Improve object recognition, reduce oxidative stress and hyperphosphorylation of tau.	
Nobiletin	APP-SL 7-5 Tg mice	Reduce Aβ deposition in hippocampus and decrease insoluble Aβ40 and Aβ142 levels in brain thereby inhibiting plaque formation.	Nakajima and Ohizumi (2019)
	3xTg mice	Improve cognitive impairment and reduce soluble Aβ levels in brain.	
	PD Models	PD Models	
	MPTP-induced PD model	Improve motor functions, cognitive deficits in passive avoidance and novel object recognition.
	MPP^+^-treated rat model of PD	Protect dopaminergic neurons from MPP^+^-induced toxicity by inhibiting neuroinflammation.	
Simvastatin (ROCK inhibitor)	Wistar rats treated with 3-NP (HD model)	Decrease ROCK expression, normalize iNOS protein expression and TNF-α level, reduce Bcl-2 protein expression, ameliorate astroglial activation and striatum injury score.	Ahmed et al. (2016)
AZA1 (Rac1, Cdc42)	*In vitro* screen of small molecules inhibitors based on NSC23766	Inhibit Rac1 and Cdc42 activity in cells at low micromolar concentrations.	Zins et al. (2013b)
ML141 (CID-2950007)	*In vitro* screen of small molecules inhibitors	Inhibit Cdc42 and also has selectivity against Rac1, Rab2, and Rab7.	Surviladze et al. (2010)
AZA197 (Cdc42-Dbs)	*In vitro* screen of small molecules inhibitors based on NSC23766	Block Cdc42-dependent migration.	Zins et al. (2013a)
ZINC08010136 (Rac1 inhibitor)	Virtual screening of the ZINC database using a pharmacophore model derived from the crystal structure of NSC23766 bound to Rac1.	Interfere with GEF/GTPase complex formation and Rac1 activity in cells.	Ferri et al. (2009)
ZINC69391 (Rac1)	Virtual screening, same as the previous ZINC compound	Blocked the Rac1-GEF interaction and Rac1 activity in cells.	Cardama et al. (2014)
MBQ-167 (Rac1)	Virtual screening	Inhibited Rac1 activity and blocked Cdc42 activity.	Humphries-Bickley et al. (2017)
	HeLa and Swiss 3T3 cells	Abolished stress fibers in Swiss 3T3 cells, competes with ATP.	Ishizaki et al. (2000)
	Crystallization from Sf9 insect cells	Induced-fit binding mode accommodated by a phosphate binding loop similar to ROCK-Fasudil complex, phosphate binding loop present which favors aromatic ring.	Yamaguchi et al. (2006)
Y-27632 (ROCK1 and ROCK2 inhibitor)	C57/BL6 mouse with LLCab tumors	Attenuates NF-κB due to overactivation, reduce inflammation from Cisplatin.	Zhu et al. (2019)
	Transgenic mouse model (AD)	Decreased the amount of toxic Aβ42.	Mueller et al. (2005)
	Smn2B/- mice (SMA)	Improves survival, improves maturation of NMJ, increased muscle fiber size.	Bowerman et al. (2010)
NSC23766 (Rac1 inhibitor)	Spraque–Dawley rats with Spinal Cord Injury	Increased mushroom shaped spines, reducing thin spines thereby reducing pain.	Cao et al. (2017)
	Primary hippocampal neurons from embryonic ICR mice.	Decrease APP protein levels.	Wang et al. (2009)
ZCL367 (Cdc42 inhibitor)	Lung cancer cell lines and prostate cancer cell lines.	Reduce proliferation, suppress migration and impede cell cycle progression, and reduce filopodia formation.	Aguilar et al. (2019)
Ibuprofen (RhoA inhibitor)	PC12 and B104 neuron-like cell culture.	Activate PPARγ promoting neurite elongation.	Dill et al. (2010)
	Chronic EAE induced in C57/BL6 mice	Enhance expression of BDNF, GDNF and NT-3.	Xin et al. (2015)
	Chronic EAE in C57/BL6 mice	Increase MAP2 expression, reduce CD4+T cells, macrophages and microglia with reduced inflammation. Increase NT-3, GDNF and BDNF.	Li et al. (2017)
FSD-C10	BV-2 cells	Inhibit the expression of all M1 markers but enhance M2 markers.
	APP/PS1 mouse model (AD)	Improve learning and memory, reduce Aβ42, reduce P-tau and BACE; Promote expression of synapse-associated proteins.	Gu et al. (2018)
	THP-1 monocyte, human cell line	Simvastatin and Lovastatin reduced IL-1B expression.	
3-hydroxy-3-methylglutaryl-coenzyme A reductase inhibitors (statins)	BV-2 cells	Downregulate iNOS, inhibit NADPH oxidase activation and ROS production, inhibit isoprenylation of Rho GTPases thereby reducing inflammation.	Cordle and Landreth (2005)
	SN4741 cells (PD)	Prevent the toxicity and apoptosis induced by the Aβ42	Manterola et al. (2013)
6-mercaptopurine (6-MP)	CD4+T Cell cultures	Increase the number of apoptosis T cells; Suppress CD28-induced activation of NF-kB p50/p56; Induce the expression of vav Rac1-activating GEF.	Tiede et al. (2003)
EHT1864 (Rac1 inhibitor)	C57/BL6 mouse hippocampal brain slices	Impair LTP, decreased levels of active Rac1, Impair long-term depression; alter mGluR-dependent LTD.	Martinez and Tejada-Simon (2011)
CNF1	Mouse models (AD and PD)	Enhance neurotransmission and synaptic plasticity and improve learning and memory in various behavioral tasks.	Diana et al. (2007) and Travaglione et al. (2020)
ZCL278 (Cdc42 modulator)	*In vitro* cell culture (AD)	Target Cdc42-ITSN GEF interaction and affect Cdc42- mediated cellular processes and neuronal growth cone dynamics.	Friesland et al. (2013)
Loganin	*In vitro* cell culture (PD)	Increase the expression of IGF-1R and GLP-1R leading to neurite outgrowth.	Tseng et al. (2019)
Cucurbitacin (RhoA)	*In vitro* cell culture	Anti-bacterial and anti-inflammatory activity; Irreversible clustering of actin and RhoA phosphorylation.	Boykin et al. (2011) and Zhang et al. (2014)
H1152 (ROCK2 inhibitor)	Human neuroblastoma cell line, cultured mouse neurons, and mouse model.	Reduced oligomeric tau, levels of phosphorylated tau, and caspase-cleaved tau.	Ikenoya et al. (2002) and Hamano et al. (2020)
SR3677 (ROCK2 inhibitor)	Human neuroblastoma cell line, mouse neurons, postmortem human AD tissue.	Suppressed β-site APP cleaving enzyme 1 (BACE1) enzymatic action and diminished production of Aβ.	Feng et al. (2008) and Herskowitz et al. (2013)
CASIN (Cdc42 inhibitor)	*In vitro* cell culture	Inhibits GEF binding to Cdc42; Inhibits F-actin polymerization.	Liu et al. (2019)
Secramine (Cdc42 inhibitor)	High-throughput synthesis and phenotypic screening; *in vitro*.	A RhoGDI for Cdc42 and inhibits Cdc42 binding to membranes, GTP, and effectors.	Pelish et al. (2006)

## Pharmacological Modulators of Rho Gtpases

### Small Molecules

Genetic tools have been developed to target Rho GTPases in disease progression including RNA interference, CRISPR, and ectopic expression of wild type or mutant proteins. These strategies, however, are hindered by a cell’s ability to adapt to genetic manipulations and the issue of a complete loss of function due to a genetic deletion. Small molecule strategy, on the other hand, provides a unique opportunity for specificity by testing the impacts on a single function of a protein while also allowing for a dose-response modulation. Small molecule drugs not only represent an alternative therapeutic avenue, but they also represent a rapid and pharmacological investigation of Rho GTPases and their roles in neurodegenerative diseases.

Small molecule hits can be identified from systematically screening chemical libraries (Gao et al., 2004; Shang et al., 2012; Friesland et al., 2013; Palsuledesai et al., 2018; Gray et al., 2020). Novel methods of computer-based in silico analysis, coupled with biochemical assays such as GTP binding (Aguilar et al., 2019) or flow cytometry-based assays (Palsuledesai et al., 2018), provide high-throughput identification of small molecule candidates for further investigation.

Among the Rho GTPases, RhoA stimulates cellular processes that act on its direct downstream effector, p160^ROCK^/Rho-associate kinase (ROCK1 and ROCK2; Clayton and Ridley, 2020). ROCK is a serine/threonine kinase that plays roles in cellular behavior, including the migration of cells and neurite growth. Multiple ROCK inhibitors have been discovered (Y-27632, fasudil or HA-1077, Y-39983, etc.) and the majority are categorized as Type 1 ATP competitive kinase inhibitors; meaning that the transfer of terminal phosphate from ATP to the respective substrate is blocked. Y-27632, widely studied and selective for both ROCK1 and ROCK2, competes with ATP (Davies et al., 2000; Ishizaki et al., 2000). Among many effects, Y-27632 is able to reverse the protein alterations associated with cellular stress (inflammation, mitochondrial deficiency, DNA repair, etc.) and those of the Rho GTPase and NF-kB signaling network in peripheral neurodegeneration (Yamaguchi et al., 2006; James et al., 2008; Zhu et al., 2019).

On the other hand, fasudil, another ROCK inhibitor, has been used in comparison with Y-27632. Fasudil inhibits both ROCK1 and ROCK2 as well, but with molecular differences from Y-27632 (Breitenlechner et al., 2003; Yamaguchi et al., 2006). This small molecule also inhibits protein kinase group AGC (PKA, PKG, and PKC), but has been shown to increase dendritic branching and stabilized dendritic arbors in CNS neurons (Couch et al., 2010), preventing neurodegeneration and stimulating neuroregeneration in various neurological disorders (Mueller et al., 2005). Additionally, fasudil effectively inhibits nitric oxide synthase immunoreactivity in microglia that mediated the neuroinflammatory response (Scheiblich and Bicker, 2017).

Compared with ROCK1, ROCK2 is mainly expressed in the brain and spinal cord, and its expression increases with aging (Koch et al., 2014). Therefore, several ROCK2 selective inhibitors including H1152 (Ikenoya et al., 2002) and SR3677 (Feng et al., 2008) were discovered and showed important features for studies of neurological diseases. For example, studies showed that H1152, applied side by side with Y-27632 and fasudil, reduced the oligomeric tau, which strengthened ROCK signaling as a potentially viable therapeutic route to reduce tauopathies, including AD (Hamano et al., 2020). Another example of inhibition of ROCK2 by using SR3677 suppressed β-site APP cleaving enzyme 1 (BACE1) enzymatic action and diminished production of Aβ in AD mouse brain (Herskowitz et al., 2013).

In terms of neuronal growth, ROCK activity leads to growth cone collapse and axonal retraction, suggesting inhibition of ROCK for stimulating axon initiation and increasing the size and motility of growth cone filopodia during neuronal maturation (Bito et al., 2000; Koch et al., 2018). It is believed that the inhibition of ROCK may not only prevent neurite collapse, but it also has the potential to enhance axonal regeneration and induce the reformation for collapsed growth cones (Liu et al., 2015).

ROCK inhibitors, however, are not the only means of modulating RhoA signaling. An inhibitor of RhoA, RhoB, and RhoC, Rhosin is an invaluable compound that directly targets GEFs to prevent the GTPases from binding and becoming activated. As such, applying Rhosin represents a useful method of studying the effects of Rho without affecting Rac1 and Cdc42 interactions (Shang et al., 2012).

Although pharmacological modulators of Rac1 and Cdc42 have been studied less extensively, several unique compounds have been developed. Based on the structure-function information of Rac 1 interaction with GEFs, a computer based virtual screening helped with the identification of NSC23766 as a highly soluble and membrane permeable compound (Gao et al., 2004; Nassar et al., 2006). NSC23766 proved to be a specific inhibitor of a subset of GEF binding to Rac and its activation. NSC23766 inhibited Rac1 GTP-loading without affecting Cdc42 or RhoA activity and suppressed the RacGEF, Tiam1. NSC23766 attenuated dendritic spine dysgenesis, decreased mechanical allodynia and electrophysiological signs of neuropathic pain (Tan et al., 2013; Cao et al., 2017). Most recently, strategy for virtual screening of small molecule was reviewed, and three unique targets were identified (Gray et al., 2020). ZINC08010136 interferes with the GEF/GTPase complex, ZINC69391 blocks the Rac1-GEF interaction, and MBQ-167 inhibits Rac1 activity and even blocks Cdc42 activity.

Discovery of Cdc42 modulators was hindered initially by the fast activation-inactivation cycles of the GTPase. Recently, high-throughput screening has succeeded in identifying ZCL278 as a small molecule that specifically targets Cdc42-ITSN GEF interaction and inhibits Cdc42- mediated cellular processes. ZCL278 thus provided a useful tool for research of the Cdc42 subclass of Rho GTPases in human pathogenesis, such as those of cancer and neurological disorders (Friesland et al., 2013). Additionally, more recent studies found that ZCL278 can act as a partial Cdc42 agonist in that when endogenous Cdc42 is not ligand activated, ZCL278 can activate Cdc42 (Aguilar et al., 2019; Lee et al., 2021). Compared with ZCL278, ZCL367 has emerged as a *bona fide* and selective Cdc42 inhibitor with nanomolar potency that suppressed cancer cell development. As such, it was proposed that ZCL367 can be an ideal candidate for lead compound optimization for further investigations (Aguilar et al., 2019).

Additional Cdc42 modulators have been identified that include small molecule CASIN (Cdc42 activity-specific inhibitor) in hematopoietic stem and progenitor cells (Liu et al., 2019) and Secramine in a RhoGDI dependent manner (Pelish et al., 2006). Targeting the GTP binding site, ML141 (CID-2950007) has also emerged as a selective and reversible non-competitive inhibitor of Cdc42 with low micromolar potency and selectivity against other members of the Rho family of GTPases such as Rac1 (Surviladze et al., 2010). The exciting collection of pharmacological modulators that span all three classical Rho GTPases will make it possible to explore their homeostatic modulations.

### NSAIDs

NSAIDs are well known for their anti-inflammatory properties and for their therapeutic potential in AD (Fink et al., 2018; Ozben and Ozben, 2019; Jordan et al., 2020). Among their several functions, NSAIDS appear to reduce Aβ production and its toxicity while also effectively blocking the Rho-cascade activation (Aguilar et al., 2017b).

A study by Oprea et al. (2015) sought to identify if the configuration of the compounds (i.e., enantiomers) had an effect on Rho GTPases. Through high-throughput screening, the authors identified that S-enantiomers of naproxen and ketorolac are inactive against GTPases with more than 20 other NSAIDs also lacking action against GTPases. The inhibitory effects of the R-enantiomers, however largely mimic those of Rac1 (NSC23766) and Cdc42 (CID2950007/ML141) specific inhibitors. As such, the authors concluded that specific NSAID R-enantiomers can effectively modulate Rac1 and Cdc42. This finding is particularly intriguing as it may help explain the mixed results from clinical trials using NSAIDs for AD treatment.

Prostaglandins (PGs) are produced *via* cyclooxygenases, which are enzymes that play a major role in neuroinflammation (Figueiredo-Pereira et al., 2016). A crucial aspect of neuroinflammation is the cyclooxygenase pathway that includes constitutive cyclooxygenase I (COX-1) and the inducible cyclooxygenase II (Cox-2; Figueiredo-Pereira et al., 2016). The variety of PGs created by these cyclooxygenases are blocked by NSAIDs. The most studied example of NSAIDs used to prompt neuroprotection is the family of Profens—ibuprofen and R-flurbiprofen being the main members. Ibuprofen is a nonsteroidal anti-inflammatory drug that is often used to treat inflammation and relieve pain in many disorders. It inhibits the signaling of RhoA, and recent studies show that ibuprofen can reduce the generation of Aβ42 (Dill et al., 2010). In the presence of Aβ, ibuprofen helped prevent neurite collapse and formation of stress fibers without affecting the formation of filopodia and lamellipodia (Ferrera et al., 2017).

Additionally, NSAIDs inhibit ADP- and collagen-induced platelet aggregation. It has been discovered that the use of Ibuprofen has led to inhibition of RhoA activation, enhancing axonal regeneration, as well as neuroprotection (Kopp et al., 2012). Unfortunately, R-flurbiprofen has shown to be ineffective at preventing/delaying loss of cognition or function in a sample of patients with AD (Green et al., 2009).

### Natural Products and Toxins

Rho GTPases are the targets of various natural products and toxins (Lerm et al., 2000). Several toxins including Clostridium botulinum C3 ADP-ribosyltransferase and Clostridium difficile toxin A and B, inactivate Rho GTPases through covalent modifications. These changes result in malformations of the actin cytoskeleton and impairment of GTPase signaling (Chen et al., 2015).

Large clostridial cytotoxins (Just and Gerhard, 2004) can modify Rho GTPase structure and function. The toxins target the GTPases at amino acid Thr37 in Rho or at the analogous residue Thr35 in Ras, Rac, and Cdc42. The toxins inhibit coupling of Rho GTPases to effectors and block subsequent signal transduction. Thus, large clostridial cytotoxins have a significant impact on GTPases and the underlying cytoskeleton. The resulting modification of the cytoskeleton leads to several cellular consequences such as dysregulation of neurotransmitter exocytosis, neuronal axon formation, and phagocytosis.

The list of natural toxins (i.e., Lerm et al., 2000) implicated in Rho GTPase signaling is continuously expanding leading to a great potential that it will provide new avenues for the development of new therapeutics (Maroccia et al., 2018).

The cytotoxic necrotizing factor 1 (CNF1) is a protein toxin derived from *Escherichia Coli*, and it modulates the Rho GTPase activity. CNF1 is known to direct the organization of the actin cytoskeleton by polymerizing it into stress fibers and membrane ruffling. CNF1 has been found to improve neuronal plasticity in neurological diseases (Travaglione et al., 2014). Recent evidence shows that intracerebral injection of CNF1 leads to a long-lasting activation of Rac1. This activation results in neuronal structural remodeling, and it significantly increased spine density and length in pyramidal neurons (Tantillo et al., 2018). Structurally, CNF1 acts by deamidating Rho GTPases on a specific glutamine residue inside the GTP-binding domain, which then blocks the molecule in their activated GTP-bound state. This allows CNF1 to modulate the actin cytoskeleton (Travaglione et al., 2020). Furthermore, CNF1 has been proven to stimulate an increase of cell energy production in mouse models of PD, as well as AD, epilepsy, and Rett syndrome (Travaglione et al., 2020).

While not a natural toxin, Cucurbitacins—triterpenoid compounds isolated from the Curcurbitacae plants—have been shown to induce actin aggregation and cofilin-actin rod formation (Zhang et al., 2014). These results, however, are mediated by the Gα13/RhoA/PKA/VASP pathway. Cucurbitacin IIa (CucIIa) has been reported to exhibit anti- cancer potential in addition to their conspicuous anti-bacterial and anti-inflammatory activity. Not only it induced the irreversible clustering of filamentous actin and reduced RhoA phosphorylation, but also δ-catenin, which suppressed RhoA, reduced efficacy of Cuc IIa to induce cell death, supporting the effects of Cuc IIa on activating RhoA and actin cytoskeletal signaling (Boykin et al., 2011). Since the actin cytoskeleton is central to learning and memory processes and cofilin is central to actin modulation, Cucurbitacins studies revealed a δ-catenin-RhoA-cofilin axis that may represent an additional route of therapeutic exploration. Finally, flavonoids from Diospyros kaki leaves have shown to regulate RhoA. In APP/PSI 4-month-old mice, the flavonoids improved learning and memory function through decreased RhoA activity (Ma et al., 2018).

## Applications of Rho Gtpase Modulators in Neurogenerative Diseases

### AD

RhoA/ROCK pathway has long been suggested as a potential therapeutic target in AD management. Over 170 ROCK inhibitors have been identified with some having great therapeutic potential for AD and other neurodegenerative diseases (Feng et al., 2016). Y-27632, a popular pan-ROCK inhibitor (Ishizaki et al., 2000), has been well studied as modulating RhoA/ROCK signaling in AD. However, recent attention has turned to ROCK2. ROCK2, but not ROCK1, is mainly expressed in the brain and spinal cord, and the expression increases as the brain ages (Hamano et al., 2020). Compared to Y-27632, fasudil is reported to be more selective towards ROCK2 and effectively inhibits disease severity in an AD mouse model (Yu et al., 2017). However, due to its relatively narrow safety window and poor oral bioavailability, fasudil may not be suitable for long-term use. A fasudil derivative, FSD-C10, demonstrated better safety profile when compared to fasudil (Xin et al., 2015). FSD-C10 showed therapeutic potential in AD mouse model, possibly through inhibiting the formation of Aβ42 and phosphorylation of tau and promoting the generation of synapse-associated proteins and neurotrophic factors (Li et al., 2017; Gu et al., 2018).

Li et al. (2016) investigated the molecular mechanism of *Xanthoceras sorbifolia* extract (XSE)—a traditional medicine used for treating CNS diseases in China—that has recently been shown to have anti-inflammatory, anti-HIV, and anti-tumor properties. Treatment with XSE reduced the RhoA/ROCK2 expression in AD model rats as compared to control rats thereby improving memory (Li et al., 2016). Nobiletin, a flavonoid isolated from citrus peels, was reported to inhibit Rho GTPase expression (Lee et al., 2011). Nobiletin has been used in AD models with some promising results. In several models, Nobiletin has been shown to improve memory impairment, protect against Aβ-induced memory deterioration, improve object recognition, reduce oxidative stress and hyperphosphorylation, and inhibit plaque formation (Nakajima and Ohizumi, 2019).

p75^NTR^ plays an important role in neuronal survival. In a series of studies, LM11A-31 and other derivatives of peptide mimetic ligands for p75^NTR^ show promising effects upstream of Rho GTPase signaling in neuroinflammation and neurodegeneration including AD (Yang et al., 2008, 2020; Elshaer et al., 2019).

AD pathology consists of Aβ deposits accumulating in the brain initiating a cascade of events which include: the release of inflammatory cytokines from microglia, the activation of Rac and Rho GTPases, and numerous downstream effects (including inflammatory component IL-1β) all resulting in the cognitive decline associated with the disease. Various studies have shown that cholesterol buildup contributes to Aβ deposit accumulation which, subsequently, led to the discovery that statins (a group of cholesterol reducing drugs) reduce these deposits. Thus, statins show the potential for treatment of AD. Cordle and Landreth (2005) found that statins reduce Aβ deposit thereby improving cognitive function. In addition, the statins inhibited Rac and Rho GTPases leading to a reduction of the downstream effectors’ characteristic of inflammatory responses, and a reduction of cholesterol accumulation in the brain (Cordle and Landreth, 2005).

AD pathology includes a decrease in dendritic spine density due to the activation of RhoA/ROCK2 in neurons which increases cognitive decline in patients afflicted with AD. The dendritic spine density is reduced when Aβ accumulation activates Caspase-2. Caspase-2 up-regulates RhoA/ROCK2 leading to the relocation of the dendritic spine head and the collapse of neuronal spines. Inhibition of Caspase-2, therefore, represents a potential therapeutic avenue for the treatment of AD to prevent the loss of dendritic spines and ultimately improving learning and memory in AD patients (Pozueta et al., 2013).

Relative to RhoA/ROCK pathway, the benefits of modulating Rac/Cdc42 is less clear in AD. NSC23766 is the most commonly used Rac1 selective inhibitor that prevents the Rac1GEF interaction necessary for nucleotide exchange (Gao et al., 2004). NSC23766 decreased APP and Aβ levels by negatively regulating APP gene transcription (Wang et al., 2009). Additionally, 6-mercaptopurine (6-MP), another Rac1 inhibitor that prevents Rac1 GTP loading, efficiently prevented Aβ42 peptide-induced cell death in SN4741 cells and in both primary neurons from the hippocampus and the entorhinal cortex (Tiede et al., 2003; Manterola et al., 2013). In addition to NSC23766, EHT1864, which inhibits Rac1 by promoting GTP unloading, altered APP metabolism processing by selectively inhibiting γ-secretase metabolism (Désiré et al., 2005).

However, it is reported that in mouse hippocampus Rac1 is highly expressed where glutamate receptor activation causes Rac1 to translocate to the membrane and play a critical role in long-term synaptic plasticity. Pharmacological inhibition of Rac1 by NSC23766 and EHT1864 impairs induction of hippocampal long-term plasticity in a dose-dependent manner (Martinez and Tejada-Simon, 2011). Additionally, studies showed that WAVE and Rac1 participate in the phagocytosis of Aβ42 in rat microglia (Kitamura et al., 2003). This and other studies raised the possibility that inactivation of Rac1/Cdc42 may lead to behavioral deficits and neurodegeneration in AD whereas they may also paradoxically inhibit Aβ metabolism and promote the clearance of pathogenic Aβ42.

## ALS and Spinal Muscular Atrophy (SMA)

ALS is marked by degeneration of motor neurons in the motor cortex, brain stem and spinal cord with most cases of familiar ALS arising from genetic mutations such as TDP-43, chromosome 9 open reading frame 72 (C9ORF72), superoxide dismutase (SOD1), fused in sarcoma (FUS) and ALS2 (Ghasemi and Brown, 2018).

ALS is analogous to spinal muscular atrophy (SMA), both of which exhibit actin dynamic disturbances as a central event associated with motor neuron degeneration. Early targets for SMA/ALS include the synaptic dynamics for proper function and maturation of neuromuscular junctions (NMJs). Treatment with ROCK inhibitor fasudil improves NMJ maturation, and the Rac1-dependent cytoskeletal dysregulation has proven to be a key determinant of motor neuron degeneration (Bowerman et al., 2010, 2012). This finding indicates that modulation of Rac1- dependent signaling pathways constitute a potential effective route to protect against neuronal degeneration (D’Ambrosi et al., 2014). Although modulators of Cdc42 have not been investigated directly in ALS, it is interesting to note that ALS2 encodes alsin2, which carries a DH/PH domain and elicits GEF properties potentially for all three classical members of Rho GTPases (i.e., RhoA, Rac1, and Cdc42; Hadano et al., 2001).

ALSin2 also suppresses SOD1 mutant neurotoxicity through RhoGEF domain (Kanekura et al., 2004). Further relationship of Rho GTPase with ALS can be inferred by the Rho-specific GEF and part of the Dbl family of GEFs encoded by the ARHGEF28 gene. This protein was first cloned from a mouse brain cDNA library and was named p190RhoGEF (Gebbink et al., 1997). As described above, the neural specific catenin, δ-catenin, is a Rho GDI that modulates interactions between RhoA and p190RhoGEF (Kim et al., 2008a, b). Given that δ-catenin interacts with AD protein presenilin and is implicated in many neurological disorders such as Cri-du-Chat, autism, and schizophrenia (Medina et al., 2000; Turner et al., 2015; Lu et al., 2016; Wang et al., 2016), modulators of δ-catenin interaction with p190RhoGEF may be a potential target that warrants further investigation.

ALS is a fatal neurodegenerative disorder where the final stage of the disease is characterized by respiratory failure. Current efforts to develop effective method of early detection for ALS have identified 20 top cellular pathways that include prominently the Rho GTPase signaling proteins in tissue fluids. This method not only has the potential to differentiate the different rates of disease progression (Leoni et al., 2019), but may also help therapeutically identify targets from perspective of peripheral blood system.

The gene C9ORF72 is known to modulate axonal growth and growth cone size in normal cells. However, it can have a G4C2 (GGGGCC) intronic repeat expansion which is often found in ALS patients. Mutating and/or depleting C9ORF72—to mimic the pathogenesis in ALS patients—has been found to enhance phosphorylation of cofilin—inactivating the F-actin assembly function. This mutation/deletion reduces actin dynamics in motor neurons and activates Arf6 which effectively leads to the activation of the downstream effector Rac1 (Sivadasan et al., 2016), again supporting the benefits of inhibit Rac1 activity in treating ALS.

SMA is an autosomal, neuromuscular disease caused by degeneration of motor neurons in the spinal cord. Epigenetic analysis linked SMA to genes in Rab and Rho GTPase signaling pathways. For example, changes in ARHGAP22 gene are associated with the activity of Rho GTPases, which are important regulators of vesicle formation, actin dynamics, axonogenesis, processes that could be critical for SMA development (Zheleznyakova et al., 2013). The cellular and molecular pathways dysregulated in SMA are highly dependent on the RhoA pathway when SMN gene is deleted; here, there is an increase in profilin IIa availability and increasing active RhoA, both decreasing the survival of affected mouse models. Inhibition of Rho kinase/ROCK by Y-27632 or fasudil leads to the increased survival and NMJ maturation in intermediate SMA mouse model as well as increasing muscle fiber size and an increase in the lifespan of the mice. However, this inhibition does not alter neurological phenotype (Bowerman et al., 2010) or prevent motor neuron degeneration. Nevertheless, inhibition of ROCK pathway has been found to be an attractive therapeutic model. The inhibition results in increased life span, an improvement in the health of remaining motor neurons, promotion of neuronal outgrowth, differentiation and guidance, normal muscle development, and enhanced myelination potential of Schwann cells and/or nerve capping at NMJ (Coque et al., 2014).

In contrast to the benefits of Rho GTPase inhibition in alleviating AD or ALS phenotypes in animal models, CNF1 constitutively activates RhoA, Rac1, and Cdc42, thus leading to remodeling of the actin cytoskeleton in intact cells. Interestingly, studies have found that CNF1 treatment of mice leads to: (i) rearrangement of cerebral actin cytoskeleton; (ii) enhanced neurotransmission and synaptic plasticity; and (iii) improved learning and memory (Diana et al., 2007). Although it was found that CNF1 treatment led to prolonged activation of Rac1 in comparison to that of RhoA in mice and rats, similar results obtained point to the potential complexity of how activation or inactivation of Rho GTPases may positively modify neurodegenerative diseases (Diana et al., 2007; Cerri et al., 2011; Aguilar et al., 2017b). This concept is supported by the recent studies that Rac1 selective inhibitor NSC23766 induced motor neuron apoptosis. Whereas RhoA/ROCK is overactivated, Rac1-GTP is decreased, and RhoB is redistributed in a well- established G93A mutant, human Cu, Zn-superoxide dismutase (hSOD1) ALS mouse model (Stankiewicz et al., 2020).

### FTD

FTD is a neurodegenerative disease characterized by progressive deficits in behavior, executive functioning, and language. FTD, in many cases, overlaps with other senior dementia, such as AD and PD, but also in 15–20% of ALS patients, where neurons in the prefrontal and temporal cortex would be variably affected (Ringholz et al., 2005). ALS and FTD can be viewed as divergent ends of the spectrum of a single but heterogeneous disease (Bang et al., 2015; Ghasemi and Brown, 2018).

The gene C9ORF72 has been discovered to be associated with both ALS and FTD when the gene contains a G4C2 repeat expansion. While excessive expansions in the C9ORF72 gene can lead to either ALS or FTD, the authors suggest that the differentiation between these two degenerative diseases could possibly be due to the higher number of hexanucleotides in ALS as compared to FTD for some cases (Dols-Icardo et al., 2014).

Because of the overlapping clinical features of FTD with AD and ALS, Rho GTPase dysregulation is likely intimately involved in the pathogenetic mechanism. Conceivably, the effective pharmacological modulation of AD and ALS may apply to FTD. The aforementioned inhibitors targeting downstream effectors of Rho GTPase signaling, such as fasudil, suppress amyloid metabolism and tauopathy in AD and FTD, and they also break down TDP-43, which aggregates in some forms of FTD and most kinds of ALS. In addition, the gene product of C9ORF72 presents both Rab- and Rho GTPase GEF activity and is predicted in lysosome biogenesis, vesicular trafficking, autophagy, and mechanistic target of rapamycin complex1 (mTORC1) signaling. Therefore, C9ORF72 may function as a dual exchange factor coupling physiological functions such as cytoskeleton modulation and autophagy with endocytosis (Iyer et al., 2018).

### PD

PD is a complex and widespread neurodegenerative disease characterized by depletion of midbrain dopaminergic (DA) neurons. PD pathology consists of characteristic intracellular LB inclusions containing α-Syn aggregates. Familial PD are linked to different mutations in LRRK2, Parkin E3-ubiquitin ligase, and PTEN-induced putative kinase 1 (PINK1), among others (Paisán-Ruiz et al., 2013; Pickrell and Youle, 2015).

PD associated LRRK2 gene mutation G2019S in Ser/Thr kinase domain leads to the delay of “damaged mitochondrial removal” or mitophagy. This delay results in neuronal cell death in vulnerable neurons, a hallmark of PD pathology. Normal mitophagy occurs when damaged mitochondria initiate the translocation of PINK1/Parkin to the affected mitochondria leading to its clearance. A study of the Miro GTPase complex revealed two Miro proteins (Miro1 and Miro2) where Miro1 induces Parkin translocation to damaged mitochondria whereas Ca^2+^ sensors initiate Miro dissociation and mitophagy machinery (Safiulina et al., 2019).

Miro is an atypical Rho GTPase and expressed in the outer mitochondrial membrane, anchoring microtubule fiber to healthy mitochondria. Damage to mitochondria results in PINK1 and Parkin relocation in conjunction to affected mitochondria. This relocation effectively leads to a degradation of Miro causing the damaged mitochondria to be removed by lysosomal mitophagy. However, the mutated LRRK2 prevents Miro degradation in damaged mitochondria. A recent discovery of small molecule Miro1 Reducer showed that it can rescue the delayed mitophagy phenotype in PD fibroblasts. Treating patient-derived neurons and fly models with this compound also rescued the locomotor deficits and dopaminergic neurodegeneration (Hsieh et al., 2016, 2019). Additional evidence for a significant role of Rho GTPases in PD pathogenesis showed that Rac1/Ced-10 activation increases autophagic clearance of α-Syn thereby preventing toxicity and increasing DA neuron survival in PD patients (Kim et al., 2018).

The dysfunctions in the insulin-like growth factor 1 (IGF-1) or glucagon-like peptide 1 (GLP-1) was reported to contribute to progressive DA neuron loss in PD. Loss of either of these proteins can lead to an increase of downstream RhoA effectors (ROCKII, p-LIMK and p-cofilin) causing cell death and subsequent loss of DA neurons. Loganin, one of the best-known iridoid glycosides, is a potential therapeutic treatment that has increased the expression of IGF-1R and GLP-1R. Loganin treatment leads to neurite outgrowth by promoting p-AKT and increasing BDNF, preventing caspase-3 cleavage, and preventing apoptotic cascade activation. In addition, Loganin inhibits RhoA translocation to the membrane down-regulating the downstream RhoA effectors. Thus, Loganin activates many neural protective survival cascades in DA neurons (Tseng et al., 2019).

With regard to the Rho GTPase activator CNF1, it increased the branching and size of dopaminergic processes. This activation led to a correction of motor asymmetry in a mouse model for an extended period after treatment, suggesting modulation of Rho GTPase pathways as a potential therapy for PD patients (Musilli et al., 2016).

In terms of DA neuronal growth, ROCK activity leads to growth cone collapse, axonal retraction, and stress fiber formation, which supports that the inhibition of ROCK stimulates axon initiation and increases the size and motility of growth cone filopodia during neuronal maturation (Koch et al., 2018). Therefore, the inhibition of ROCK may not only prevent neurite collapse, but it also has the potential to enhance axonal regeneration and induce the reformation for collapsed growth cones (Fu et al., 2018). In a mouse model of PD, inhibition of ROCK enhances survival of DA neurons and attenuates axonal loss (Tönges et al., 2012).

### Others (HD and MS)

HD is a progressive neurological disease consisting of motor and psychiatric impairments by neuronal dysregulation and loss of neurons in cortex and striatum. Pathogenesis of this disease is characterized by toxic properties of mutated huntingtin (HTT) resulting in loss of function. HD signaling pathways showed highest concentration of dysregulated genes. Primary pathogenic mechanisms of HD link HTT to signaling complexes including Rho GTPases in dynamic organization of neuronal processes and cell-cell contacts (Tourette et al., 2014). The hallmark of HD pathogenesis involves motor disturbances in basal ganglia dysfunction as a result of deficits of dendritic spine structure, location, and number involved in synaptic functions. There are increasing data which provide evidence for a specific coupling of D2R-Short but not D2R-Long isoform to the RhoA/ROCK/cofilin pathway, and its involvement in striatal vulnerability to expanded HTT. Thus, a new route for targeting Rho-ROCK signaling in HD is unraveled (Galan-Rodriguez et al., 2017).

As discussed earlier, Kalirin-7 is a RacGEF and is essential for maintenance and branching of dendritic spines. The inhibition of Rac1 GTPase and Kalirin-7 contributes to cortico-striatal dysfunction in HD model mice leading to the inhibition of excitatory synapses in HD pathology. Interestingly, overexpression of Kalirin-7 restores excitatory synapses in neuronal cortical cultures (Puigdellívol et al., 2015), indicating the potential of activating Rac1 as a therapeutic option.

MS is an inflammatory, demyelinating disease of CNS and a major cause of neurological disability. The pathology of this disease includes inflammation, demyelination of neurons, oligodendrocyte death, and axonal degeneration. Rho GTPases play an integral part and suggest as a promising target for treatment. Treatment with ROCK inhibitor Y-39983, a more potent derivative of Y-27632, revealed a faster recovery from disease, decreased clinical symptoms, decreased inflammatory lesions, and decreased demyelination. This treatment led to an increase in myelinated axons and an increased complete recovery in MS model mice with experimental autoimmune encephalomyelitis or EAE (Gao et al., 2013).

In addition, MS studies have shown that mitochondrial dysfunction contributes to the disease mechanisms. Singh et al. (2018) tested the combination of lovastatin and AICAR (an analog of adenosine monophosphate, or AMP), which is capable of stimulating AMP-dependent protein kinase (AMPK) activity, as potential treatments resulting in a reduction of overall pathology. AICAR suppressed RhoA while implicated the activation of Rac1 (Sha et al., 2014). Likewise, lovastatin led to inhibition of RhoA/ROCK activity, protection of AMPK activity in spinal cords, reparation of mitochondrial defects, reduction of clinical symptoms, and reduction of CNS disease pathogenesis (Singh et al., 2018). Statins contain anti-inflammatory properties by inhibiting CNS lesion formation. They contain neuroprotective and neuro-reparative properties by inhibiting demyelination of axons and/or remyelination of axons *via* the RhoA pathway leading to a promising treatment option for MS patients (Ciurleo et al., 2014).

## Challenges and Conclusion

Increasing evidence suggests that neurodegenerative diseases are multifaceted and that Rho GTPase modulation, at least in part, showed promises to attenuate the effects of related pathology. Recent studies have helped to elucidate the therapeutic potential of Rho GTPases, but the exact mechanisms of some pharmacological tools remain unclear. Throughout this review, multiple examples of Rho GTPase dysregulation and their respective modulation have been presented and help portray the delicate balance of Rho GTPase activity. Due to the complexity of Rho GTPase signaling, an understanding of each of the major Rho GTPase (e.g., RhoA, Rac1, Cdc42) signaling cascades are necessary to effectively evaluate their therapeutic potential. As noted, it is also important to determine the role of Rho GTPase regulators (e.g., GEFs, GAPs, and GDIs) as such mechanisms have not yet been sufficiently addressed. Even more, crosstalk between Rho GTPases and other molecular regulators underscores the complexity of these pathways. Another obstacle with Rho GTPase modulation *in vivo* is the translatability between animal models and the pathology of neurodegenerative diseases. New animal models that better represent human pathology and can address the shortfalls of earlier models and methodologies are critically needed.

In addition, there has been a significant increase in pharmacological tools that have been developed to help effectively screen and target potential therapeutic options. These tools help address the earlier challenges of Rho GTPase drug discovery by enabling exploration of all elements of the Rho GTPase signaling cascade. As such, the elucidation of basic mechanisms, the creation of greater translational models, and the development of more effective screening tools make pharmacologically targeting Rho GTPases ever closer to therapeutic application in neurodegenerative diseases.

Rho GTPases serve diverse roles as signaling proteins within cells of the nervous system. Genetic mutations of the genes that encode for Rho GTPases as well as mutations that interrupt the normal functioning of their modulators can lead to many diseases including neurodegenerative disorders. The modulations of Rho GTPases have great translational potential due to Rho GTPase’s strong implications in these human diseases. Meanwhile, recent progresses are also accompanied by the recognition of complex Rho GTPase modulation where targeting its signaling can improve some aspects of pathogenesis while exacerbating others in the same disease model. Therefore. much is still to be elucidated on how different Rho GTPases work in concert and how they produce such widespread yet selective cellular responses during disease progression.

## Author Contributions

WG, AK, CB, and QL wrote the article, held discussions, and finalized article. WG drafted figure and table. CB revised table. QL conceptualized the article and guided the writing project. All authors contributed to the article and approved the submitted version.

## Conflict of Interest

The authors declare that the research was conducted in the absence of any commercial or financial relationships that could be construed as a potential conflict of interest.
